# Is social support related to better mental health, treatment continuation and success rates among individuals undergoing in-vitro fertilization? Systematic review and meta-analysis protocol

**DOI:** 10.1371/journal.pone.0252492

**Published:** 2021-06-01

**Authors:** Marisa Casale, Anna Carlqvist

**Affiliations:** 1 Department of Social Policy and Intervention, University of Oxford, Barnett House, Oxford, United Kingdom; 2 School of Public Health, University of the Western Cape, Bellville, South Africa; University of Sorocaba, BRAZIL

## Abstract

Infertility and its treatment via in-vitro fertilization (IVF) represent a global health area of increasing importance. However, the physical and psychological burden of IVF can negatively impact psychological wellbeing, as well as treatment retention and success. Social support has been found to have positive health effects among populations facing health-related stressors worldwide, and its potential protective role for IVF patients merits further attention. We present a protocol for a systematic review of peer-reviewed published studies quantitatively investigating associations between social support and i) mental health; ii) the decision to (dis)continue with IVF treatment cycles and; iii) IVF success (pregnancy and birth rates); among individuals who are undertaking or have undertaken IVF cycles. Studies will be included if they work with human subjects, provide correlation coefficients between measures of social support and at least one of the outcomes of interest, and are in the English language. Social support may derive from both naturally occurring networks and more formalized sources or interventions. The protocol for this systematic review was developed according to the PRISMA-P guidelines. Ten health-, psychology- and sociology-related databases will be searched using composite search terms that include keywords for ‘IVF’ and ‘social support’. To assess methodological quality, the authors will use a modified version of the Newcastle-Ottawa Scale. Should three or more moderate or good quality studies be identified for any one outcome of interest, correlation meta-analyses, using the Hedges-Olkin method, will be conducted to pool effect sizes and heterogeneity will be assessed. Should the number, quality and characteristics of eligible studies not allow for reliable quantitative synthesis, the authors will limit the analysis to qualitative synthesis, with a focus on implications of findings for future research and programming.

## Introduction and rationale

Infertility and its treatment represent a global health area of increasing importance. Individuals experiencing infertility represent around 8–10% of couples worldwide [1–3]. The demand for assisted reproduction techniques such as in-vitro fertilization (IVF) has increased in developed countries over the past decades, and is predicted to increase further in those to come [2, 4]. This could be further boosted by numbers in resource-limited settings of the developing world, where an estimated 180 million couples are experiencing infertility [5–7].

The efficacy of IVF among the assisted fertility options has led an increasing number of individuals to seek this specific treatment. More than a half million babies are now born each year from IVF, as a result of over two million estimated annual treatment cycles [8, 9]. Yet it is also a relatively invasive and disruptive process that can be both physically and psychologically demanding [10, 11]. Infertility itself has been associated with a higher prevalence of depression and anxiety, lower quality of life, and lower self-esteem [11, 12]. The IVF process–which includes injectable medication and multiple blood tests, clinic appointments and procedures, waiting periods and anticipation of outcomes at each phase—may lead to further psychological stress. This can be exacerbated by disturbances to an individual’s work and routine and the financial pressure of this costly treatment [13, 14], and may be worse after multiple failed cycles [10, 15, 16].

Higher levels of stress and worse mental health have in turn been shown to be associated with lower odds of pregnancy in general and specifically within the context of assisted fertility [17–19], suggesting that this could potentially translate into a vicious cycle for infertile couples who have experienced multiple unsuccessful cycles. Moreover, the treatment dropout or discontinuation rates for fertility treatments are often high, as a result of psychological, physical and financial burden related to these procedures [20–22]. This ultimately reduces the odds of success since multiple IVF cycles are often necessary to achieve a live birth [8, 23].

Considering the potential immediate and long term psychological effects of infertility and IVF, described above, the role of social support as a protective resource for individuals undergoing IVF merits further attention. Qualitative research with IVF patients has, in fact, exposed patients’ desire for greater emotional advice and support, and professional psychosocial services, even where individuals are satisfied with the medical care received [24–27]. Moreover, a recent review on the psychological consequences of IVF argued for further studies investigating the effect of supportive social interactions for the functioning of couples undergoing these procedures [26]. Social support has been shown to be protective for mental and physical health outcomes, and to influence health behaviours (such as retention in healthcare) among numerous populations around the world [28, 29]. Adequate support, particularly emotional support, may contribute to protecting the mental health of individuals undergoing IVF, and potentially increasing the odds of continuing with treatment cycles and achieving a positive outcome.

The few systematic reviews published to date on related topics are either dated and do not include papers published after 2014 [30, 31], do not include a quantitative synthesis of findings [30] and/or focus on infertility or assisted fertility more generally, versus specifically IVF [19, 31, 32]. None include all three outcomes of psychological health, IVF success and treatment continuation. Moreover, two of these reviews synthesise studies assessing efficacy of psychosocial interventions on psychological or pregnancy outcomes [19, 32], which is distinct from assessing associations with measurable dimensions of social support.

Yet investigating support deriving both from interventions and naturally occurring relationships is important, considering that a large number of individuals undergoing IVF do not have access to formal support interventions, or may choose not to participate in or complete these [19, 33]. Also, to date, besides some evidence supporting approaches such as cognitive behavioural therapy (CBT) and group psychotherapy, most intervention evaluations do not show evidence of impact on mental health or live births among IVF patients [19, 33].

## Review questions

We will conduct a systematic review of studies to identify, synthesize and discuss the published evidence to date addressing the following questions:

1. Is social support associated with psychological wellbeing among individuals undergoing IVF?
2. Is social support associated with IVF success rates?
3. Is social support associated with the decision to continue with IVF treatment?

Our aim is to address these three questions quantitatively through meta-analysis, should the number, quality and characteristics of eligible studies allow for quantitative synthesis. Should meta-analysis not be possible, we will synthesize the evidence on these phenomena qualitatively with a focus on programmatic and research implications.

## Methodology

The protocol for this systematic review was developed according to the Preferred Reporting Items for Systematic Review and Meta-Analysis Protocols (PRISMA-P) [34].

### Study participants

Study participants will be only human subjects. Specifically they will be individuals and/or couples seeking to undergo, undergoing or who have undergone in vitro fertilisation.

### Intervention

Our ‘intervention’ or exposure for this review is social support, as defined above. ‘Social support’ is a multi-dimensional construct that may include the existence, quantity and type of interpersonal relationships (network structure or social interaction), the functional content of social relationships (e.g. types of support provided) and the perceived quality or adequacy of this support [35–37]. The functional aspects of social support are distinct from, but also linked to, the structural aspects of support. The amount and types of support accessible or received will ultimately depend on an individual’s structural ties, including social network size and the type of relationships [38]. It should, however, be noted that functional dimensions of support have been found to be more strongly associated with better mental health outcomes than structural aspects of support [39, 40]. These functions, or “types” of support, are most frequently categorized as emotional (e.g., love, care, and encouragement), instrumental (e.g., providing tangible items or practical assistance), and informational [36].

Sources of social support include an individual’s network of ‘naturally occurring’ relationships [41], e.g. with family or friends, or more ‘formalised’ sources, such as support interventions linked to health facilities and delivered by healthcare workers, counsellors or other professionals. Eligible studies should include one or more measurable dimension of social support.

### Outcomes

For question 1) the primary outcomes will be mental health constructs, including anxiety, depressive mood and psychological distress, likely measured through standardized psychometric tools. For question 2) the primary outcomes will be rates of successful pregnancy and, if available, live birth rates. For question 3) the primary outcome will be the decision to continue or discontinue with IVF treatment. Based on the number, quality and characteristics of studies included, potential confounding factors or moderators to be explored in meta-regression could include: study location, the gender of participants, participant age, the type of construct used for social support, the type of specific outcome (e.g. anxiety versus depression) and study design (for example, whether cross-sectional or longitudinal).

### Inclusion criteria

This systematic review will identify studies quantitatively assessing associations between one or more dimensions of social support and: i) mental health outcomes; ii) IVF ‘success’ outcomes, namely pregnancy and live birth rates and/or; iii) the decision to continue with IVF treatment cycles; among individuals who have undertaken or are undertaking an IVF cycle. Studies will only be included if they provide correlation coefficients between measures of social support and at least one of the outcomes of interest, or sufficient information for these to be calculated. No limitation will be imposed for the commencement date of published articles, although a clear cut-off date of 30 June 2021 will be applied. The review will include cross-sectional and longitudinal correlational studies.

In the case of publications deriving from the same study and sample, all papers will be included in the table and narrative synthesis, although the distinction between number of papers and number of studies will be made clear. Should we proceed to quantitative synthesis of the papers, the publication with the largest sample size or the first published study (if sample sizes are the same) will be included in the analysis. Conference papers, dissertations and reviews will not be included. The search will be limited to peer-reviewed publications and English-language studies, given the absence of resources for translation.

### Timeline

The systematic review and its write-up will be completed within approximately 6 months of finalisation and publication of this review protocol.

## Review methods

Ten health-, psychology- and sociology-related databases (listed in S1 Table) will be searched; these include Pubmed and Medline. A keyword search strategy and composite search term have been developed according to the Cochrane Collaboration PICOS inclusion criteria [42], as illustrated in S1 Table. The same keywords will be used in all databases but the search strings will be adjusted to fit with the criteria for each database. To minimize the risk of excluding relevant papers, we will not include keywords for specific mental health or IVF outcomes of interest nor for type of intervention, but will limit search terms to only include keywords for ‘IVF” and ‘social support’ (the population and intervention/phenomenon). An example search string is included in S1 Table.

The authors will screen the titles and abstracts independently to determine relevance. Full text papers will then be downloaded for potentially relevant abstracts and assessed by both authors. Discrepancies will be resolved through re-assessment and discussion. Additionally, the citation lists of all papers that meet the review inclusion criteria will be searched to identify further potentially relevant papers. Endnote software will be used to support the management of records, full text and duplicates. A flow chart with results of the review will be presented by means of a Prisma diagram [34].

A template will be created in Microsoft Excel as a data extraction tool. This standardized form will be used to extract key information and characteristics of each study. S2 Table lists the data items to be extracted from the studies. These items are informed by the Replication and Transparent Reporting of Evaluations with Nonrandomized Designs checklists, as well as other published systematic review protocols in this field [43, 44]. Extracted data will include: study design, recruitment methods, sample size and key characteristics, constructs and measurement tools used for social support and the outcomes of interest. The authors will make adjustments to this tool after entering the first few papers, through discussion and agreement. Each author will extract the data independently and discrepancies will be resolved through discussion.

## Quality assessment methods

Both authors will independently review the eligible studies for methodological quality, and resolve discrepancies in the assessments through discussion. This review will apply a modified version of the Newcastle-Ottawa Scale [45] as a quality assessment tool; this tool has been used in previous social science systematic reviews [46, 47] as it allows for the assessment of a wide range of study designs. The Newcastle-Ottawa Scale tool assesses criteria related to participant selection, exposure to the intervention (where relevant), comparability (appropriate adjustment for confounders), assessment of outcomes and other potential sources of bias. A template for the presentation of assessments through this tool is included in S4 Table. Each study will be allocated a final quality of evidence rating of ‘low’, ‘moderate’ or ‘good’ quality, based on the assessment of overall risk of bias. A judgment of ‘UNCLEAR’ will be made for individual risk of bias items within these tools, where the study report does not provide adequate information.

## Data synthesis and meta-analysis methods

The final relevant papers included in the review will be organized and presented in table format (see S3 Table for an example of what this will look like). Studies will likely be organised based on the outcomes investigated and the study design. Information drawn from the extraction sheet and presented in the final tables will include: the publication dates, the dates and location of research conducted, the sample sizes and characteristics, the study design and methodology, the specific constructs and indicators used to measure social support and the outcomes of interest and the correlation coefficients.

### Narrative data synthesis

A narrative synthesis will be used to qualitatively describe the included studies, according to selected characteristics and categories in the extraction table. Findings of studies will be grouped and presented based on each outcome of interest, i.e. mental health, treatment continuation and IVF success rates respectively. We will highlight the number of studies investigating the relationship between social support and each outcome, and indicate how many and which studies found significant associations. We will describe the constructs and psychometric tools used to measure social support, including the type and sources of support these refer to, where applicable. We will similarly describe the tools and constructs used for specific outcomes within each outcome category (e.g. depression and anxiety for mental health).

### Correlation meta-analysis

Should there be more than 3 studies assessed to be of moderate or good quality for any one outcome of interest (mental health, IVF success or treatment continuation), we will conduct a correlation meta-analysis to pool effect sizes for the associations between social support and the specific outcome. Since study samples are likely to be drawn from different populations, we will run a random effects model and test for heterogeneity. Based on the number and quality of studies, as well as the level of heterogeneity, we will determine whether this analysis is reliable.

We will use the Hedges-Olkin method, based on a conventional summary meta-analysis with a Fisher transformation of the correlation coefficient [48]. This entails calculating a Fisher’s Z-value for each correlation, as well as the variance of the correlation. A pooled correlation and variance will be calculated with the transformed Z values and results will be back transformed to the original scale or *r*. Should zero-order correlation coefficients not be reported in the papers, an attempt will be made to contact the first authors to obtain these. The meta-analysis will be conducted using a software package that supports pooling of correlation coefficients, such as STATA version 13.0 or MedCalc (https://www.medcalc.org/index.php).

To assess heterogeneity, or the variation in outcomes between studies, we will consider indicators such as the τ2, Q and the I² statistics. The I² statistic in particular summarizes the inconsistency of results across studies, which is the percentage of variation across studies that is due to heterogeneity rather than chance [49]. The heterogeneity indicators will be considered together with a qualitative assessment of the appropriateness of combining studies. Drawing from the *Cochrane Handbook for Systematic Reviews* indications, an I² statistic between 0% and 30% will be interpreted as an insignificant amount of heterogeneity; 30% to 60% will represent moderate heterogeneity; 60% to 100% will be considered as representing substantial heterogeneity [42]. A low I² would suggest that a fixed effects model might be appropriate. A moderate or high I² is more likely and would instead confirm the appropriateness of a random effects approach to estimate pooled effect measures, which is considered to be the more natural choice by many health investigators [50–52].

### Meta-regression and subgroup analysis

If we identify a sufficient number of studies (10 or more) for a given predictor per outcome category, we will use meta-regression and subgroup analysis to explore sources of heterogeneity and their likely influence on pooled measures of effect. Given sufficient studies, and based on the information provided, we will conduct random-effects meta-regression analysis [50] including predictors such as: different sources or types of social support dimensions assessed (e.g. structural versus functional indicators; emotional versus instrumental support; different key support providers); different outcome constructs (e.g. depression versus anxiety for mental health); and participant characteristics such as age and gender. Given sufficient studies, we will also conduct separate subgroup analyses by study design to determine, for example, whether there are differences for cross-sectional versus longitudinal studies [10, 53–55]. We will determine whether to proceed with sensitivity analysis, and which analyses to conduct, once individual study characteristics and data shortcomings are identified during the review process.

### Assessment of publication bias

Studies with high effect sizes are more likely to be published than those with low effect sizes, leading to publication bias and an over-estimation of the pooled effect in our meta-analysis [56]. If we are able to pool 10 or more studies, we will create a funnel plot to visually explore the risk of publication bias, interpreting the results with caution [57]. An asymmentrical funnel would indicate the presence of publication bias, suggesting that only small studies with a large effect size have been published while small studies with small effect sizes are missing.

### Ethics and dissemination of findings

This study will not require ethics approval by specific Ethics Boards since it will only involve synthesis of published secondary data, and there will be no primary data collection with human subjects. The results of this systematic review will be published in the form of a peer-reviewed journal article. They will also be presented to various audiences including academics, practitioners, development agencies through scientific conferences, potential short policy documents, stakeholder meetings and through the authors’ networks.

## Potential strengths, limitations and unique contribution of this review

This review will have a number of methodological strengths. The search will be developed and conducted by two experienced researchers, one with over a decade of experience in research on social support and health, and the other a medical doctor with experience in public health research. The study selection and data extraction will be performed independently by the two researchers and widely used tools will be employed to assess the studies’ methodological quality.

This review will also have various limitations. These may include an insufficient number and quality of eligible studies to run meta-analysis for some or all of the outcomes of interest. Moreover, while we posit social support to be a protective resource for the outcomes of interest, based on existing literature, we cannot determine causality from non-randomized correlational studies. Should the included studies be predominantly cross-sectional, this will further limit our ability to infer causality of the associations between social support and the outcomes assessed. Also, the inclusion of only peer-reviewed papers may exclude grey literature containing relevant analyses; however, limiting the review to peer-reviewed papers will also insure a minimum quality of studies.

Despite its limitations, this review has the potential to make a valuable contribution to the existing literature in this area. To our knowledge this would be the first review to aggregate the recent evidence on associations between social support–deriving from naturally occurring relationships or more formalized sources–and mental health, IVF treatment continuation and success rates among IVF patients specifically. Findings will potentially highlight whether social support should be considered and further investigated as a health-promoting resource or protective factor for individuals undergoing fertility treatments, for mental health, treatment continuation and IVF success. Should measurement indicators of social support indicate specific types and/or sources of support, a combination of narrative synthesis and potential subgroup analyses could highlight which particular types or sources of support may be particularly important for practitioners working with individuals undergoing IVF. For example, should the review expose a positive relationship between ‘informal’ social support (from social networks and relationships) and the outcomes of interest, this would highlight the potential for intervention developers and implementers to involve members of their beneficiaries’ informal networks in support interventions or potentially work to strengthen these networks. It may also highlight the types of support that should be targeted (e.g. emotional versus instrumental).

Conversely, findings of a positive relationship between social support and the outcomes of interest may also indicate whether patients with less social support are potentially more at risk for less favourable mental health and IVF outcomes. These individuals may need to be specifically targeted during potential pre-treatment screening processes [58], and possibly referred to support programs or more specialized psychosocial support.

Lastly, this review will aim to expose gaps in the existing literature, to inform the foci and design of future studies investigating the relationship between social support, mental health and treatment retention and success, among individuals and couples undergoing IVF treatment.

## Supporting information

S1 Table Systematic review search strategy. (DOCX)

S2 Table Information to be extracted from eligible studies. (DOCX)

S3 Table Characteristics of studies included in the review. (DOCX)

S4 Table Methodological quality and risk of bias assessment template. (DOCX)

S1 Checklist PRISMA-P 2015 checklist. (DOCX)
