# Supplementary material for: Is social support related to better mental health, treatment continuation and success rates among individuals undergoing in-vitro fertilization? Systematic review and meta-analysis protocol
Source: PLoS One. 2021 Jun 1;16(6):e0252492. doi: 10.1371/journal.pone.0252492 (PMC8168841; doi:10.1371/journal.pone.0252492)
Supplement: S1 Table — (DOCX) [file pone.0252492.s001.docx]

| **S1 Table.** **Systematic review search strategy** | |
| --- | --- |
| **Search criteria** (as per the PICOS inclusion criteria; Cochrane Collaboration) | **P**opulation: individuals who are undergoing or have undergone in vitro fertilization (IVF) treatment for infertility  **I**ntervention: Social support, deriving from naturally occurring relationships or more formalized sources/interventions  **C**omparison: N/A  **O**utcomes: mental health outcomes; IVF success as measured by outcomes such as pregnancy and live birth; treatment (dis)continuation  **S**tudy design: studies quantitatively assessing associations between social support and the outcomes of interest; no further limitations |
| **Databases to search** | Academic search complete (ASC); CINAHL; Health Source: Nursing and Academic Edition; Medline; Psychology and Behavioral Science Collection (PBS); PsychINFO; SocINDEX; Cochrane online; Pubmed; Proquest Health and Medical Complete |
| **Example of composite search string** (for databases searched through Ebscohost) | (“in vitro fertilization” OR “in vitro fertilisation” OR “in-vitro fertilization” OR “in-vitro fertilisation” OR IVF OR “assisted reproduct* OR “assisted fertility” OR “assisted conception” OR “fertility treatment”)  AND (“social support” OR “support network” OR “support intervention” OR “social network” OR “psychosocial support” OR “psycho-social support” OR “support program*” OR “emotional support” OR “social relation*” OR “social interaction” OR “partner support” OR “friend support” OR “family support” OR “husband support” OR “wife support” OR “marital support” OR “partnership support” OR “couple* support”) |
| **Limiters** | Peer-reviewed publications  English language  Published before 30 June 2021 |
