# Supplementary material for: Is social support related to better mental health, treatment continuation and success rates among individuals undergoing in-vitro fertilization? Systematic review and meta-analysis protocol
Source: PLoS One. 2021 Jun 1;16(6):e0252492. doi: 10.1371/journal.pone.0252492 (PMC8168841; doi:10.1371/journal.pone.0252492)
Supplement: S2 Table — (DOCX) [file pone.0252492.s002.docx]

| S2 Table. Information to be extracted from eligible studies |
| --- |
| Title of paper |
| Paper Authors |
| Publication year |
| Country and setting where the research was conducted |
| Study design |
| Participant inclusion criteria and recruitment |
| Number of participants initially recruited in the study |
| Number of participants who completed the study |
| Key participant characteristics: e.g. age, gender |
| Duration of study (for longitudinal designs) |
| Methods and timing of data collection |
| Constructs and measurement tools used for social support |
| Outcomes of interest assessed |
| Measurement tools used to assess outcomes |
| Correlation coefficients and confidence intervals |
