# Supplementary material for: Is social support related to better mental health, treatment continuation and success rates among individuals undergoing in-vitro fertilization? Systematic review and meta-analysis protocol
Source: PLoS One. 2021 Jun 1;16(6):e0252492. doi: 10.1371/journal.pone.0252492 (PMC8168841; doi:10.1371/journal.pone.0252492)
Supplement: S3 Table — (DOCX) [file pone.0252492.s003.docx]

| **S3 Table. Characteristics of studies included in the review** | | | | | | | | |
| --- | --- | --- | --- | --- | --- | --- | --- | --- |
| **Citation** | **Location** | **Study design** | **Methods and timing of data collection** | **Participants** | **Social support construct and measures** | **Outcomes and measures** | **Correlation coefficients** | **Quality score** |
| *Study authors and year of publication* | *Country and setting in which the research was conducted* | *Overall study design (e.g. cross-sectional, prospective cohort)* | *Methods used for data collection, number of data collection waves, dates or time periods for each wave of data collection* | *Number, gender and other specific characteristics of participants included in the final analyses* | *Constructs and measurement tools used for social support* | *Outcome(s) assessed in the study (e.g. depression, anxiety) and* *measures used for each of these.* | *Correlation coefficients between social support and the outcomes of interest, with confidence intervals.* | *Outcome of NOS quality assessment* |
