# Supplementary material for: Is social support related to better mental health, treatment continuation and success rates among individuals undergoing in-vitro fertilization? Systematic review and meta-analysis protocol
Source: PLoS One. 2021 Jun 1;16(6):e0252492. doi: 10.1371/journal.pone.0252492 (PMC8168841; doi:10.1371/journal.pone.0252492)
Supplement: S4 Table — (DOCX) [file pone.0252492.s004.docx]

| **S4 Table. Methodological quality and risk of bias assessment template** | | | | | | | | | | | |  |
| --- | --- | --- | --- | --- | --- | --- | --- | --- | --- | --- | --- | --- |
| Study | Participant selection |  | Exposure to intervention |  | Comparability | Assessment of outcomes |  |  |  | Other potential sources of bias |  | |
|  | **Were participants selected to be representative of the target population?** | **Were there clear participant selection criteria avoiding inappropriate exclusions?** | **Was a comparator/control group assessed?** | **Was the intervention being studied applied consistently to all eligible participants?** | **Were potential confounders identified and appropriately adjusted for?** | **Were procedures for assessment of outcome sufficient to satisfy confirmation of presence of condition of interest?** | **Was follow-up long enough for outcome to occur?** | **Were incomplete outcome data adequately addressed?** | **Are reports of the study free of suggestion of selective outcome reporting?** | **Was the study free of other problems that could put it at a high risk of bias?** | **Overall quality of study** | |
| *1. Study citation* |  |  |  |  |  |  |  |  |  |  |  | |
